# Follicular Bronchiolitis Associated With Primary IgG2/IgG4 Deficiency in a Previously Healthy 40-Year-Old Woman

**DOI:** 10.7759/cureus.22183

**Published:** 2022-02-13

**Authors:** Mansur Assaad, Anam Aqeel, James Walsh

**Affiliations:** 1 Pulmonary and Critical Care Medicine, Guthrie Robert Packer Hospital, Sayre, USA; 2 Internal Medicine, Guthrie Robert Packer Hospital, Sayre, USA

**Keywords:** interstitial lung disease, immunodeficiency, tree-in-bud, igg2/igg4 deficiency, follicular bronchiolitis

## Abstract

Follicular bronchiolitis (FB) associated with immunodeficiency is not commonly reported in peer-reviewed literature. Herein, we present a case of FB associated with IgG2/IgG4 deficiency. The presence of non-specific respiratory symptoms, including cough and dyspnea with exertion, led to a CT scan of the chest, which showed diffuse, peripheral, micronodular tree-in-bud opacities and an isolated area of bronchiectasis. Despite an extensive workup, including a non-diagnostic transbronchial biopsy, no obvious etiology for the patient’s clinical presentation was established, and a surgical lung biopsy was needed to confirm the diagnosis of FB. Further lab testing to evaluate for immunodeficiency confirmed an isolated deficiency in both IgG2 and IgG4. Although treating the underlying cause of FB is the standard of care, there are no established guidelines regarding standard management of FB associated with immunodeficiency, specifically IgG2/IgG4 deficiency. Therefore, a careful evaluation for immunodeficiency should be considered when evaluating for the underlying etiology of FB, as management options differ depending on the underlying diagnosis.

## Introduction

Follicular bronchiolitis (FB) is a rare lymphoproliferative pulmonary disease characterized by hyperplasia of the bronchial-associated lymphoid tissue (BALT) [1]. It is theorized that antigenic stimulation of the BALT results in polyclonal lymphoid hyperplasia and the development of lymphoid follicles with germinal centers in the walls of small airways [1]. FB is classified based on etiology, and common causes include primary (idiopathic), familial, connective tissue disease, immunodeficiency, infections, interstitial lung disease, and airway inflammatory disorders [2]. The most common immunodeficiencies related to FB are acquired immunodeficiency syndrome (AIDS) and common variable immune deficiency (CVID) [2]. Although patients with IgG2/IgG4 deficiency are at increased risk for sinopulmonary infections, rhinosinusitis, and bronchitis, an association with FB is not well established in peer-reviewed literature.

## Case presentation

**Figure 1:**
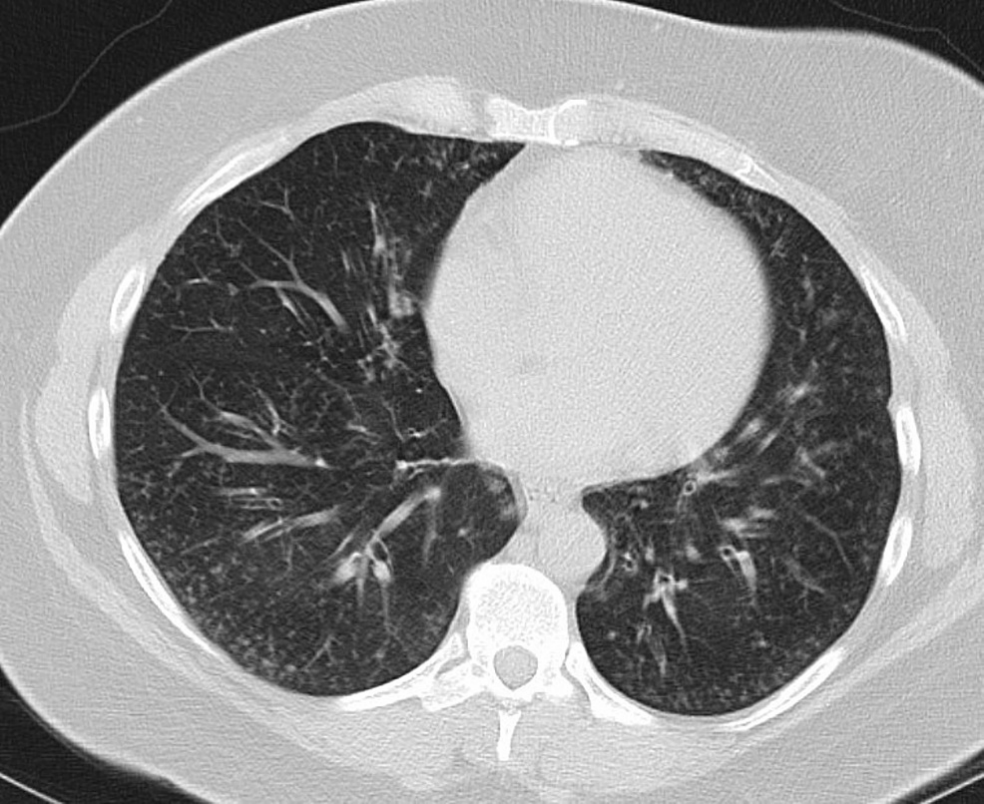
Axial CT image showing multiple bilateral centrilobular and tree-in-bud nodules, concerning for small airway disease.

A 40-year-old woman with type 1 diabetes mellitus and a 24 pack-year history presented with chronic dyspnea on exertion and productive cough for the past four months. Computed tomography (CT) of the chest showed multiple bilateral centrilobular and tree-in-bud nodules and right upper lobe (RUL) bronchiectasis (Figure 1).

**Figure 2:**
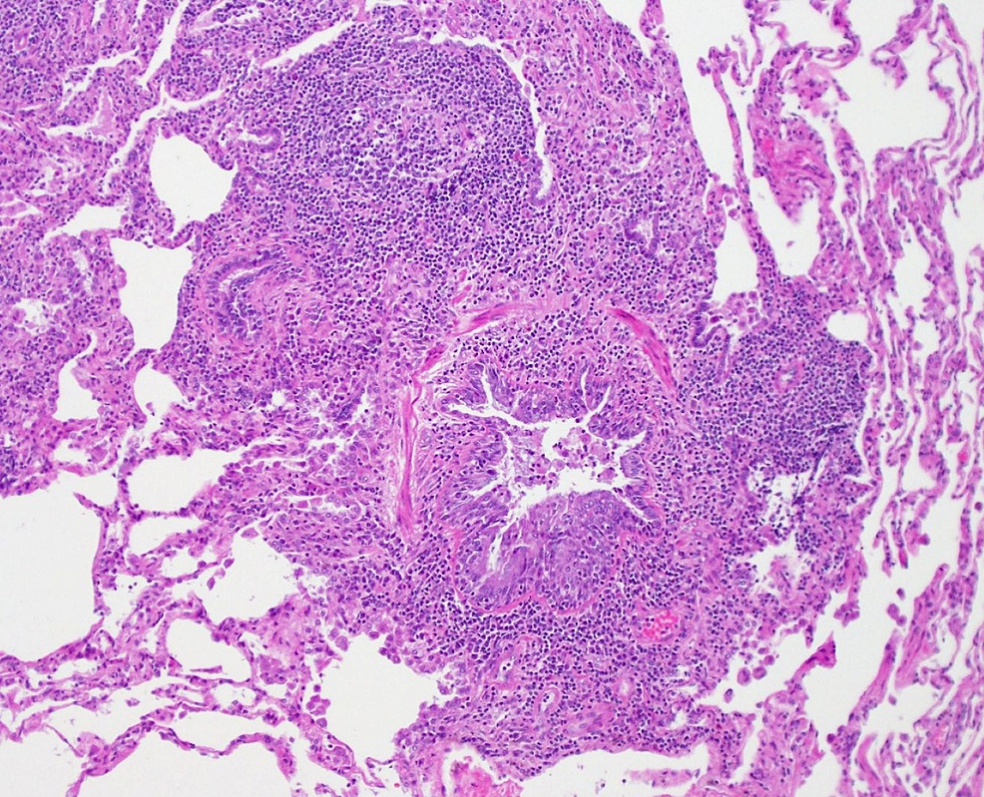
Hematoxylin and eosin (H&E) stain from surgical lung biopsy showing airway narrowing with enlarged peribronchiolar lymphoid follicles, consistent with follicular bronchiolitis.

Pulmonary function tests (PFTs) showed a mixed pattern with restriction and moderately severe airflow obstruction with no significant reversibility. A bronchoscopy was performed. Culture from the bronchoalveolar lavage of the RUL grew *Haemophilus influenzae* and methicillin-sensitive *Staphylococcus aureus*. Transbronchial biopsy of RUL showed focal non-necrotizing epithelioid granulomatous inflammation. Fungal and mycobacterial cultures showed no growth. Despite antibiotics and empiric treatment for sarcoidosis with prednisone, the patient’s symptoms did not resolve and repeat CT showed no radiographic improvement. Extensive workup for connective tissue disease, rheumatic disease, hypersensitivity pneumonitis, and fungal infection was unremarkable. Complete blood count showed no leukocytosis or peripheral eosinophilia. Repeat routine sputum culture and three separate mycobacterial sputum cultures showed no growth. The interferon-gamma release assay was negative. Human immunodeficiency virus testing was negative. Quantitative immunoglobulin levels showed normal IgG, IgM, and IgA levels. However, further IgG subclass testing revealed a decreased IgG2 level at 190 mg/dL and IgG4 level at 0.9 mg/dL. Additionally, the immunoglobulin E (IgE) level was elevated at 497 kU/L, but this was felt to be non-specific. Ultimately, a surgical lung biopsy was obtained, and the final pathology showed bronchiolar lumen narrowing with surrounding enlarged lymphoid follicles, consistent with FB (Figure 2).

She was started on azithromycin 250 milligrams (mg) daily. On follow-up, both symptoms and PFT findings improved significantly (Table 1).

**Table 1:** Pulmonary function tests before and after treatment. FEV1, forced expiratory volume at one second; FVC, forced vital capacity; TLC, total lung capacity; DLCO, diffusion capacity of the lung for carbon monoxide; N/A, not applicable.

	Lower limit of normal	Initial	Two months after starting treatment
FEV1 (L)	2.63	1.81	2.50
FVC (L)	3.26	2.23	3.14
FEV1/FVC (%)	73	66	80
TLC (L)	4.38	4.25	N/A
DLCO (mL/min/mmHg)	18.46	15.98	22.63

## Discussion

The etiology of FB can generally be classified as either primary or secondary [2]. Secondary FB is more common, and further classification can be made depending on the underlying systemic or pulmonary disorder [2]. Common connective tissue diseases that are associated with secondary FB include Sjogren’s syndrome, rheumatoid arthritis, and systemic lupus erythematosus [2]. One must also consider infectious etiologies, particularly *Pneumocystis jirovecii*, *Legionella pneumonia*, and acute viral hepatitis [2]. Certain interstitial lung diseases have been associated with FB, including lymphoid interstitial pneumonia, respiratory bronchiolitis-associated interstitial lung disease, desquamative interstitial pneumonia, cryptogenic organizing pneumonia, and hypersensitivity pneumonitis [2,3]. Finally, certain immunodeficiencies may predispose patients to the development of FB, particularly CVID and AIDS [2,3]. Given the wide range of possible etiologies, a thorough diagnostic workup is required to narrow the differential diagnosis. With our patient, preliminary lab work for evaluating connective tissue disease, recurrent infections, and hypersensitivity pneumonitis was unremarkable. Although HIV testing was negative and total immunoglobulin levels were normal, an isolated deficiency in IgG2 and IgG4 raised the suspicion for primary IgG2/IgG4 deficiency leading to recurrent bacterial pulmonary infections possibly associated with the development of FB. To the best of our knowledge, this is the first reported patient of secondary FB associated with IgG2/IgG4 deficiency.

Commonly reported clinical manifestations include dyspnea, cough, and recurrent pneumonia [4]. However, the clinical presentation of FB may vary depending on the severity of any underlying systemic disease. For example, in patients with underlying connective tissue disease, symptoms related to any underlying systemic rheumatic disease tends to predominate early in the disease course with progressive dyspnea developing later insidiously thereafter [4]. Patients with underlying immunodeficiency tend to present in teenage years or early adulthood with progressive dyspnea and recurrent sinopulmonary infections [4]. Pulmonary function tests are non-specific and may show a normal, obstructive, restrictive, or mixed pattern [5]. Our patient had moderate restriction and notable airflow obstruction on initial PFTs, and spirometric values improved significantly after starting treatment.

Common CT findings include micronodules with a “tree-in-bud” pattern, patchy ground-glass infiltrates, air trapping, mosaic attenuation, and reticular infiltrates [4]. Our patient displayed some of these characteristic findings, including diffuse micronodules with a tree-in-bud pattern, which likely represents bronchial obstruction with inflammatory exudate and peribronchial lymphoid follicle formations. With regards to histopathological diagnosis, the yield from bronchoscopy with transbronchial biopsy is low, and surgical lung biopsy is usually required [2,5]. In our case, transbronchial biopsy results were misleading, as the presence of non-necrotizing granulomas led to an incorrect preliminary diagnosis of sarcoidosis. With regards to secondary FB, management is aimed at treating the underlying etiology. Patients with connective tissue disease often respond well to targeted immunosuppressant therapy aimed at managing the underlying primary disorder [2]. Patients with HIV often respond well to proper management with antiretroviral therapy [2]. Replacement immunoglobulin therapy has been shown to reduce the frequency and severity of pulmonary infections associated with CVID; however, no guidelines exist regarding immunoglobulin replacement therapy in managing FB associated with IgG2/IgG4 deficiency [2]. Macrolide antibiotics have been used to manage patients with primary FB, and improvement in symptoms is likely related to the anti-inflammatory properties of macrolides [2]. Our patient was successfully managed with chronic azithromycin therapy with a significant reduction in symptom severity and improvement in PFT findings noted on follow-up.

## Conclusions

FB is a rare form of interstitial lung disease with several potential underlying etiologies. Since image and PFT findings are often non-specific, a formal diagnosis requires correlation with clinical, radiographic, and histopathological findings. Further evaluation for immunodeficiency, infection, and connective tissue disease is required, as treatment differs greatly depending on the underlying etiology.
